# Association of serum growth differentiation factor 11 with asthma severity, inflammation, and airway remodeling in pediatric patients

**DOI:** 10.3389/fmed.2026.1872411

**Published:** 2026-07-20

**Authors:** Yanran Che, Ji Yang

**Affiliations:** Department of Pediatrics, Shanghai Pudong New Area People's Hospital, Shanghai, China

**Keywords:** airway remodeling, asthma exacerbation, biomarker, GDF11, inflammation, pediatric asthma

## Abstract

**Background:**

Growth differentiation factor 11 (GDF11), a member of the transforming growth factor beta (TGF-*β*) superfamily, is implicated in tissue remodeling and inflammation. However, its role in asthma, particularly during exacerbations remains unclear. This study investigated the association between serum GDF11 levels and asthma severity, inflammatory responses, and airway remodeling in pediatric patients.

**Methods:**

This cross-sectional study enrolled 107 children with asthma (72 with stable asthma and 35 with acute exacerbations) and 110 age- and sex-matched healthy controls. Pulmonary function parameters, inflammatory biomarkers, airway remodeling-related factors, and serum GDF11 levels were assessed. Correlation analyses and multivariate logistic regression were performed to evaluate the association between serum GDF11 levels and clinical characteristics. Receiver operating characteristic (ROC) analysis was used to assess the diagnostic performance of GDF11 in identifying asthma exacerbations.

**Results:**

Compared with healthy controls, children with asthma exhibited significantly lower serum GDF11 levels (*p* < 0.001), which were further reduced during exacerbations (*p* < 0.001). Multivariate analysis identified higher GDF11 levels as being independently associated with reduced odds of exacerbation (odds ratio [OR] = 0.879, 95% confidence interval [CI]: 0.776–0.995, *p* = 0.041). Serum GDF11 levels were positively correlated with pulmonary function parameters (FEV1: *r* = 0.387, FEV1/FVC: *r* = 0.681, PEF: *r* = 0.357; all *p* < 0.001) and inversely correlated with eosinophil counts, total immunoglobulin E (IgE), fractional exhaled nitric oxide (FeNO), inflammatory cytokines (IL-1β, IL-4, IL-6, TNF-*α*), and airway remodeling markers (TGF-β1, SDF-1α, MMP-2, MMP-9) (all *p* < 0.05). ROC analysis indicated that serum GDF11 distinguished asthma exacerbation with good accuracy (AUC = 0.839, sensitivity = 77.6%, and specificity = 77.3%).

**Conclusion:**

Reduced serum GDF11 levels are associated with asthma exacerbations, airway inflammation, and remodeling in pediatric patients. GDF11 may serve as a potential biomarker of asthma severity and exacerbation risk and is associated with airway inflammatory and structural changes in children with asthma.

## Background

Asthma is a chronic inflammatory airway disease characterized by episodic airflow obstruction, bronchial hyperresponsiveness, and airway remodeling, which involves the thickening of the reticular basement membrane, smooth muscle hypertrophy, fibroblast activation, and extracellular matrix (ECM) deposition (1, 2). These structural alterations contribute to persistent airflow limitation and disease severity in subgroups of patients and are driven by complex interactions between epithelial cells, immune cells, and mesenchymal effectors in response to environmental and immunological stimuli (3, 4). Airway remodeling is a central pathophysiological feature of asthma that is closely linked to chronic inflammation and acute exacerbations of disease symptoms (5, 6).

The transforming growth factor-*β* (TGF-β) superfamily plays a pivotal role in regulating inflammation and remodeling responses in patients with asthma. Among its members, canonical TGF-*β* isoforms (TGF-β1, TGF-β2, and TGF-β3) are elevated in asthmatic airways and have been shown to promote subepithelial fibrosis, airway smooth muscle proliferation, mucous cell hyperplasia, and epithelial-mesenchymal transition (EMT) through Smad2/3-dependent and non-Smad signaling pathways (7, 8). Increased transforming growth factor beta-1 (TGF-β1) expression correlates with disease severity and airway wall thickening and is considered a central driver of structural remodeling in asthma (9, 10). Although the profibrotic and immunomodulatory roles of TGF-*β*1 and related cytokines have been extensively investigated, the contribution of other TGF-β superfamily members, such as growth differentiation factor 11 (GDF11), to chronic airway inflammation and remodeling remains poorly understood (11–13).

GDF11, also known as bone morphogenetic protein 11 (BMP11), is a circulating cytokine of the TGF-*β* superfamily with diverse biological effects, ranging from embryonic development to the regulation of inflammation and tissue remodeling (14, 15). While initial studies highlighted its potential “rejuvenating” effects in aging tissues, including the heart and skeletal muscle (12, 16), subsequent research has revealed a more complex and context-dependent role for GDF11 in tissue homeostasis and disease (11). Mechanistically, GDF11 signaling predominantly involves activin type II receptors (ActRIIA and ActRIIB) and downstream activation of the canonical Smad2/3 pathways (13), which overlap with the intracellular signaling mechanisms implicated in inflammatory regulation and fibrotic remodeling across multiple organ systems (17).

Recent evidence supports a dual role for GDF11 in inflammation and tissue injury in different organs. For example, GDF11 has been shown to exhibit anti-inflammatory effects in experimental models of colitis and arthritis, whereas in lung tissue, it can act as a pro-inflammatory and profibrotic agent, inducing epithelial injury, fibroblast activation, and extracellular matrix deposition through ALK5-Smad2/3 signaling (18, 19). These findings suggest that GDF11 may contribute to airway structural changes analogous to those observed in chronic fibrotic lung diseases and underscore the need to explore its role in airway inflammation and remodeling.

Despite the recognized involvement of TGF-*β* signaling in the pathogenesis of asthma and airway remodeling, the specific association between serum GDF11 levels and the clinical features of asthma, including acute exacerbations, inflammation, and structural airway changes, remains poorly defined. To address this knowledge gap, the present study investigated the relationship between circulating GDF11 concentrations and markers of acute exacerbations, airway inflammation, and asthma remodeling. Elucidating this association may help clarify the clinical significance of GDF11 in chronic airway diseases and inform the development of novel biomarkers for disease assessment in the future.

## Materials and methods

### Study population

This cross-sectional study was conducted in the Department of Pediatrics at our hospital between July 2023 and June 2025. A total of 107 children with asthma were enrolled. All baseline clinical and laboratory measurements were obtained at the index visit before the initiation of any asthma-specific treatment. None of the participants were receiving regular maintenance controller therapy, including inhaled corticosteroids or leukotriene receptor antagonists at the time of enrollment.

Asthma was diagnosed based on typical clinical symptoms and confirmed variable expiratory airflow limitation in accordance with the Guidelines for the Diagnosis and Treatment of Childhood Bronchial Asthma (2016) (20). Exacerbation was defined as the acute onset or worsening of respiratory symptoms (e.g., wheezing, shortness of breath, chest tightness, and cough) accompanied by reduced expiratory airflow. Based on clinical status at enrollment, children with asthma were classified into a stable asthma group (*n* = 72) or an asthma exacerbation group (*n* = 35). In addition, 110 age- and sex-matched healthy children undergoing routine physical examinations were recruited as controls.

**Figure 1:**
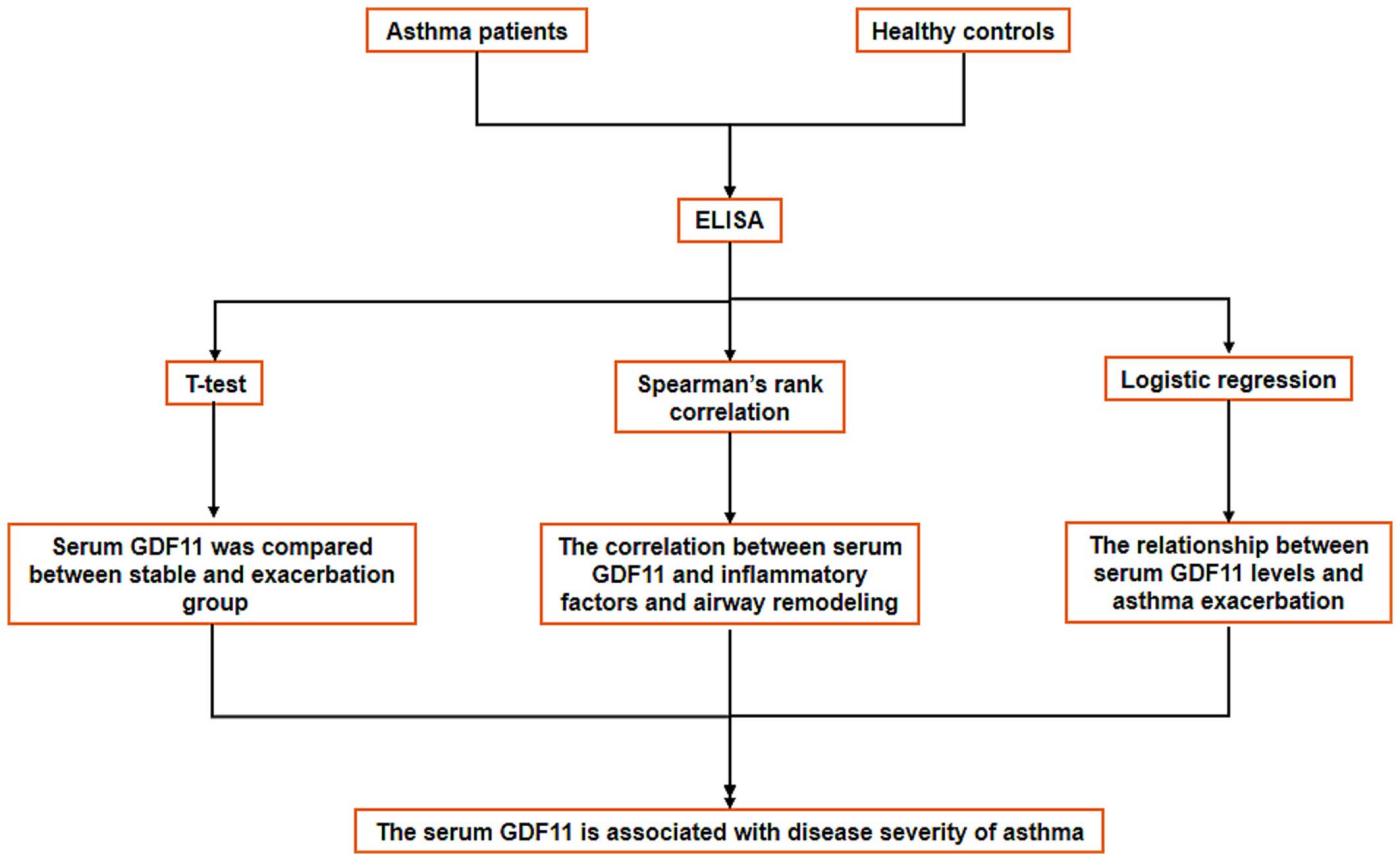
Clinical research flowchart. This study is a cross-sectional comparison of children with stable asthma and those with asthma exacerbations, based on their clinical status at enrollment. A total of 107 pediatric patients with asthma and 110 healthy controls were included in the study. Clinical, pulmonary function, inflammatory, and airway remodeling parameters were assessed and compared between the groups. Spearman’s rank correlation and multivariate logistic regression analyses were performed.

Although participants were contacted during routine clinical follow-up at approximately 3 and 6 months after discharge for general monitoring, these visits were not part of a predefined prospective protocol, and standardized data on exacerbations, lung function, or biomarker reassessment were not systematically collected. Consequently, complete and analyzable longitudinal outcome data were unavailable for a substantial proportion of participants. Therefore, the present study was designed and analyzed as a cross-sectional comparison based exclusively on baseline clinical and laboratory data collected at enrollment, and no longitudinal outcomes were evaluated. Figure 1 summarizes the study design, variables assessed, and analytic workflow. This study was approved by the Medical Ethics Committee of our hospital.

Given the diagnostic challenges in younger children (≤5 years), for whom spirometry may be unreliable, diagnostic classification in this subgroup was additionally supported by comprehensive clinical assessment, including symptom patterns, treatment response, fractional exhaled nitric oxide (FeNO), and immunological markers, such as serum total IgE levels and peripheral blood eosinophil counts.

### Exclusion and inclusion criteria

The inclusion criteria were as follows: (i) age 3–12 years; (ii) clinically diagnosed asthma based on typical clinical symptoms and confirmed variable expiratory airflow limitation according to established diagnostic guidelines, with disease duration recorded at enrollment based on symptom onset; (iii) no prior long-term standardized asthma maintenance therapy within the preceding 3 months, including inhaled corticosteroids or leukotriene receptor antagonists; (iv) complete clinical data; and (v) good cooperation and compliance during examinations.

The exclusion criteria were as follows: (i) presence of other respiratory system diseases; (ii) comorbid diseases involving other major organ systems, such as the heart, liver, or kidneys; (iii) history of other chronic allergic or immunological diseases that may confound the results; (iv) immunodeficiency disorders; and (v) use of systemic glucocorticoids or other medications affecting immune or inflammatory status within the past 3 months.

### Clinical data collection

After admission, clinical data were collected from each pediatric patient with asthma, including age, sex, body mass index (BMI), and duration of asthma. The duration of asthma reported in this study reflects the time from symptom onset to study enrollment after clinical diagnosis. Approximately 5 mL of fasting venous blood was collected from all pediatric patients before treatment upon admission. White blood cell (WBC) count, lymphocyte, neutrophil, eosinophil, and total immunoglobulin E (IgE) levels were measured. FeNO levels were measured using a nitric oxide analyzer (Sunvou-CA2122 system, China) (21). Pulmonary function was measured in all subjects, including forced expiratory volume in one second (FEV1), forced vital capacity (FVC), and peak expiratory flow rate (PEF). The FEV1 relative to forced vital capacity (FEV1/FVC) were calculated.

### Fractional exhaled nitric oxide (FeNO)

Two hours before the FeNO testing, the child should avoid vigorous exercise or eating nitrogen-containing foods such as animal organs and spinach. The child should sit in a seated position, place the nasal plug in one nostril, and use a portable nitric oxide analyzer (NIOX VERO®; Aerocrine, Solna, Sweden) to continuously aspirate nasal gas for 6 s to obtain the data (22). The tests were repeated 3 times to reduce variability, and the mean was used as the final test result.

### Enzyme-linked immunosorbent assay (ELISA)

Fasting venous blood samples were collected from all children, centrifuged at 3000 r/min for 10 min to obtain serum and stored at −20 °C. An enzyme-linked immunosorbent assay (ELISA) was used to measure the serum levels of interleukin-1 beta (IL-1β; DLB50, R&D Systems), interleukin-4 (IL-4; D4050, R&D Systems), interleukin-6 (IL-6; D6050B, R&D Systems); tumor necrosis factor-alpha (TNF-*α*; SEKH-0047, Solarbio), TGF-β1 (SEKH-0316, Solarbio), stromal cell-derived factor 1-alpha (SDF-1α; ml038196, Shanghai Enzyme-linked Biotechnology), matrix metalloproteinase-2 (MMP-2; MMP200, R&D Systems), matrix metalloproteinase-9 (MMP-9; DMP900, R&D Systems), and GDF11 (SEKH-0111, Solarbio) in the serum of all study participants.

### Statistical analysis

Continuous variables are expressed as mean ± standard deviation (SD), and categorical variables as frequency (percentage). Independent-sample t-tests were used to compare continuous variables between the asthma and healthy control groups, as well as between the stable asthma and asthma exacerbation groups. Categorical variables were analyzed using the χ^2^-test. Receiver operating characteristic (ROC) curve analysis was performed to determine the optimal cutoff value for serum GDF11 levels to discriminate between children with asthma and healthy controls. Spearman’s rank correlation coefficient was used to assess associations between serum GDF11 (and periostin) levels and pulmonary function indices, blood routine parameters, immune indicators, inflammatory cytokines, and airway remodeling markers, given the non-normal distribution of these variables. Multiple logistic regression analysis was conducted to identify risk factors for asthma exacerbation. To evaluate the potential influence of body mass on the observed associations, BMI was included as a covariate in a sensitivity analysis of the multivariate logistic regression model assessing risk factors for asthma exacerbation. Because none of the enrolled patients received maintenance controller therapy at baseline, medication use was not included as a covariate in the multivariate logistic regression analysis. All statistical analyses were performed using baseline clinical, functional, and laboratory data obtained at enrollment; no longitudinal outcomes, including subsequent exacerbations or changes in the lung function, were included in this analysis. Statistical analyses were conducted using SPSS version 30.0, and a two-sided *p* value < 0.05 was considered statistically significant.

## Results

### Clinical and laboratory characteristics of the study population

As summarized in Table 1, 110 healthy controls and 107 patients with asthma were included in this study population. No significant differences were observed between the asthma and healthy control groups with respect to age, sex distribution, or BMI (asthma: 21.84 ± 2.64 kg/m^2^; controls: 22.04 ± 2.61 kg/m^2^; all *p* > 0.05). Pulmonary function indices, including FEV1, FVC, FEV1/FVC ratio, and PEF, were significantly reduced in the asthma group compared to the control group (all *p* < 0.001). Patients with asthma exhibited higher white blood cell, eosinophil, and neutrophil counts, and lower lymphocyte percentages (all *p* < 0.001). Serum IgE levels and FeNO were markedly elevated in the asthma group (*p* < 0.001). Inflammatory cytokines (IL-1β, IL-4, IL-6, TNF-*α*, and TGF-β1) were significantly increased in patients with asthma (all *p* < 0.001). The levels of SDF-1α, MMP-2, and MMP-9 were also higher in the asthma group (*p* ≤ 0.002). In contrast, circulating GDF11 levels were significantly lower in patients with asthma than in healthy controls (*p* < 0.001).

**Table 1:** Characteristics of the healthy controls and asthma patients.

Variables	Control group (*n* = 110)	Asthma group (*n* = 107)	*p*-value
Age (years)	7.35 ± 2.06	7.21 ± 2.10	0.644
Sex (male, %)	69 (62.7%)	65 (60.7%)	0.764
BMI (kg/m^2^)	22.04 ± 2.61	21.84 ± 2.64	0.563
FEV1 (L)	2.29 ± 0.48	1.77 ± 0.37	<0.001
FVC (L)	2.73 ± 0.57	2.38 ± 0.43	<0.001
FEV1/FVC (%)	84.08 ± 4.32	74.18 ± 4.85	<0.001
PEF (L/min)	310.49 ± 29.07	248.20 ± 34.74	<0.001
WBC (per/μL)	7420.99 ± 1067.47	8512.73 ± 1201.63	<0.001
Lymphocyte (%)	37.67 ± 6.63	32.12 ± 5.76	<0.001
Neutrophil (%)	50.80 ± 9.23	55.90 ± 9.25	<0.001
Eosinophil (per/μL)	276.85 ± 60.39	530.62 ± 108.32	<0.001
IgE (IU/mL)	144.53 ± 26.54	265.78 ± 48.30	<0.001
FeNO (ppb)	13.86 ± 2.61	44.57 ± 12.88	<0.001
IL-1β (pg/mL)	96.06 ± 18.09	121.77 ± 20.32	<0.001
IL-4 (pg/mL)	68.84 ± 8.54	78.96 ± 10.54	<0.001
IL-6 (pg/mL)	17.38 ± 2.14	22.05 ± 3.22	<0.001
TNF-α (pg/mL)	20.95 ± 5.71	33.60 ± 5.28	<0.001
TGF-β1 (pg/mL)	38.80 ± 5.74	45.36 ± 7.94	<0.001
SDF-1α (pg/mL)	465.80 ± 64.06	496.27 ± 75.78	0.002
MMP-2 (ng/mL)	158.51 ± 30.43	175.78 ± 34.74	<0.001
MMP-9 (ng/mL)	108.16 ± 19.83	132.81 ± 24.65	<0.001
GDF11 (ng/mL)	47.70 ± 6.57	37.71 ± 7.51	<0.001

Continuous variables are expressed as mean ± standard deviation (SD), and are analyzed by t test or Wilcoxon-Mann–Whitney test. Categorical variables are expressed as frequency (percentage), and are analyzed by chi-square test.

BMI, body mass index; FEV1, forced expiratory volume in one second; FVC, forced vital capacity; PEF, peak expiratory flow rate; WBC, white blood cell; IgE, immune globulin E; FeNO, exhaled nitric oxide; IL-1β, interleukin 1β; IL-4, interleukin 4; IL-6, interleukin 6; TNF-α; tumor necrosis factor-α; TGF-β1, transforming growth factor-β1; SDF-1α, stromal-derived factor 1α; MMP-2, matrix metalloproteinases-2; MMP-9, matrix metalloproteinases-9; GDF11, growth differentiation factor 11.

### Clinical characteristics of patients with stable asthma and asthma exacerbation

As shown in Table 2, patients with stable asthma (*n* = 72) and asthma exacerbation (*n* = 35) did not differ significantly in terms of age, sex distribution, or body mass index (all *p* > 0.05). The duration of asthma was significantly longer in patients with exacerbations than in those with stable disease (*p* < 0.001). Pulmonary function was more severely impaired in the exacerbation group, as evidenced by significantly lower FEV1, FEV1/FVC ratio, and PEF (all *p* ≤ 0.007), whereas FVC did not differ between the groups (*p* = 0.148). Patients with asthma exacerbation exhibited higher eosinophil counts, serum IgE levels, and FeNO levels than those with stable asthma (all *p* ≤ 0.008). Lymphocyte percentage was modestly increased in the exacerbation group (*p* = 0.022), whereas total WBC count and neutrophil percentage were comparable between groups. Inflammatory mediators, such as IL-1β, IL-4, and TGF-β1, were significantly elevated during exacerbation (all *p* ≤ 0.033), whereas IL-6 and TNF-*α* levels showed no significant differences. The levels of SDF-1α, MMP-2, and MMP-9 were significantly higher in the exacerbation group (all *p* ≤ 0.013). In contrast, circulating GDF11 levels were markedly reduced in patients with asthma exacerbations compared to those with stable asthma (*p* < 0.001).

**Table 2:** Characteristics of patients with stable asthma and asthma exacerbation.

Variables	Stable asthma (*n* = 72)	Asthma exacerbation (*n* = 35)	*p*-value
Age (years)	7.03 ± 1.99	7.60 ± 2.30	0.188
Sex (male, %)	42 (58.3%)	23 (65.7%)	0.463
BMI (kg/m^2^)	21.91 ± 2.69	21.69 ± 2.59	0.687
Duration of asthma (years)	2.19 ± 0.52	2.80 ± 0.58	<0.001
FEV1 (L)	1.85 ± 0.39	1.62 ± 0.28	<0.001
FVC (L)	2.42 ± 0.46	2.30 ± 0.36	0.148
FEV1/FVC (%)	76.13 ± 4.37	70.17 ± 2.98	<0.001
PEF (L/min)	254.41 ± 35.63	235.41 ± 29.37	0.007
WBC (per/μL)	8399.64 ± 1195.35	8745.37 ± 1197.91	0.164
Lymphocyte (%)	31.24 ± 5.29	33.94 ± 6.32	0.022
Neutrophil (%)	55.33 ± 9.67	57.06 ± 8.34	0.368
Eosinophil (per/μL)	496.24 ± 93.94	601.34 ± 102.48	<0.001
IgE (IU/mL)	257.25 ± 47.84	283.32 ± 44.98	0.008
FeNO (ppb)	40.99 ± 12.07	51.95 ± 11.37	<0.001
IL-1β (pg/mL)	116.45 ± 18.27	132.71 ± 20.18	<0.001
IL-4 (pg/mL)	77.45 ± 10.41	82.07 ± 10.26	0.033
IL-6 (pg/mL)	21.72 ± 3.14	22.76 ± 3.31	0.116
TNF-α (pg/mL)	32.94 ± 5.10	34.94 ± 5.45	0.066
TGF-β1 (pg/mL)	43.62 ± 7.38	48.95 ± 7.95	<0.001
SDF-1α (pg/mL)	483.70 ± 70.79	522.11 ± 80.12	0.013
MMP-2 (ng/mL)	169.36 ± 33.94	189.00 ± 33.02	0.006
MMP-9 (ng/mL)	124.59 ± 21.41	148.08 ± 23.63	<0.001
GDF11 (ng/mL)	40.37 ± 6.66	32.24 ± 6.14	<0.001

BMI, body mass index; FEV1, forced expiratory volume in one second; FVC, forced vital capacity; PEF, peak expiratory flow rate; WBC, white blood cell; IgE, immune globulin E; FeNO, exhaled nitric oxide; IL-1β, interleukin 1β; IL-4, interleukin 4; IL-6, interleukin 6; TNF-α; tumor necrosis factor-α; TGF-β1, transforming growth factor-β1; SDF-1α, stromal-derived factor 1α; MMP-2, matrix metalloproteinases-2; MMP-9, matrix metalloproteinases-9; GDF11, growth differentiation factor 11.

### Multivariate analysis of factors associated with asthma exacerbation

Multivariate logistic regression analysis identified several independent predictors of asthma exacerbation (Table 3). Longer asthma duration was significantly associated with an increased odds of exacerbation (odds ratio [OR] = 4.262, 95% confidence interval [CI]: 1.390–13.065, *p* = 0.011). In contrast, a higher FEV1/FVC ratio was inversely associated with exacerbation risk (OR = 0.815, 95% CI: 0.671–0.990, *p* = 0.040). Elevated blood eosinophil counts were independently associated with a higher risk of exacerbation (OR = 1.008, 95% CI: 1.000–1.015, *p* = 0.038), and increased serum MMP-9 levels were also associated with a higher exacerbation risk (OR = 1.032, 95% CI: 1.000–1.065, *p* = 0.049). Conversely, higher circulating GDF11 levels were associated with reduced odds of asthma exacerbation (OR = 0.879, 95% CI: 0.776–0.995, *p* = 0.041). In sensitivity analyses adjusted for BMI, serum GDF11 levels remained independently associated with reduced odds of asthma exacerbation, with effect estimates comparable to those of the primary model. Overall, these findings suggest that disease duration, lung function impairment, inflammatory biomarkers, and GDF11 levels independently contribute to the risk of exacerbation.

**Table 3:** Multivariate logistic regression for asthma exacerbation of patients.

Characteristics	Odds ratio	95% confidence interval	*p*-value
Duration of asthma (years)	4.262	1.390–13.065	0.011
FEV1/FVC (%)	0.815	0.671–0.990	0.040
Eosinophil (per/μL)	1.008	1.000–1.015	0.038
MMP-9 (ng/mL)	1.032	1.000–1.065	0.049
GDF11 (ng/mL)	0.879	0.776–0.995	0.041

FEV1, forced expiratory volume in one second; FVC, forced vital capacity; MMP-9, matrix metalloproteinases-9; GDF11, growth differentiation factor 11.

### Serum GDF11 levels and diagnostic performance in asthma

**Figure 2:**
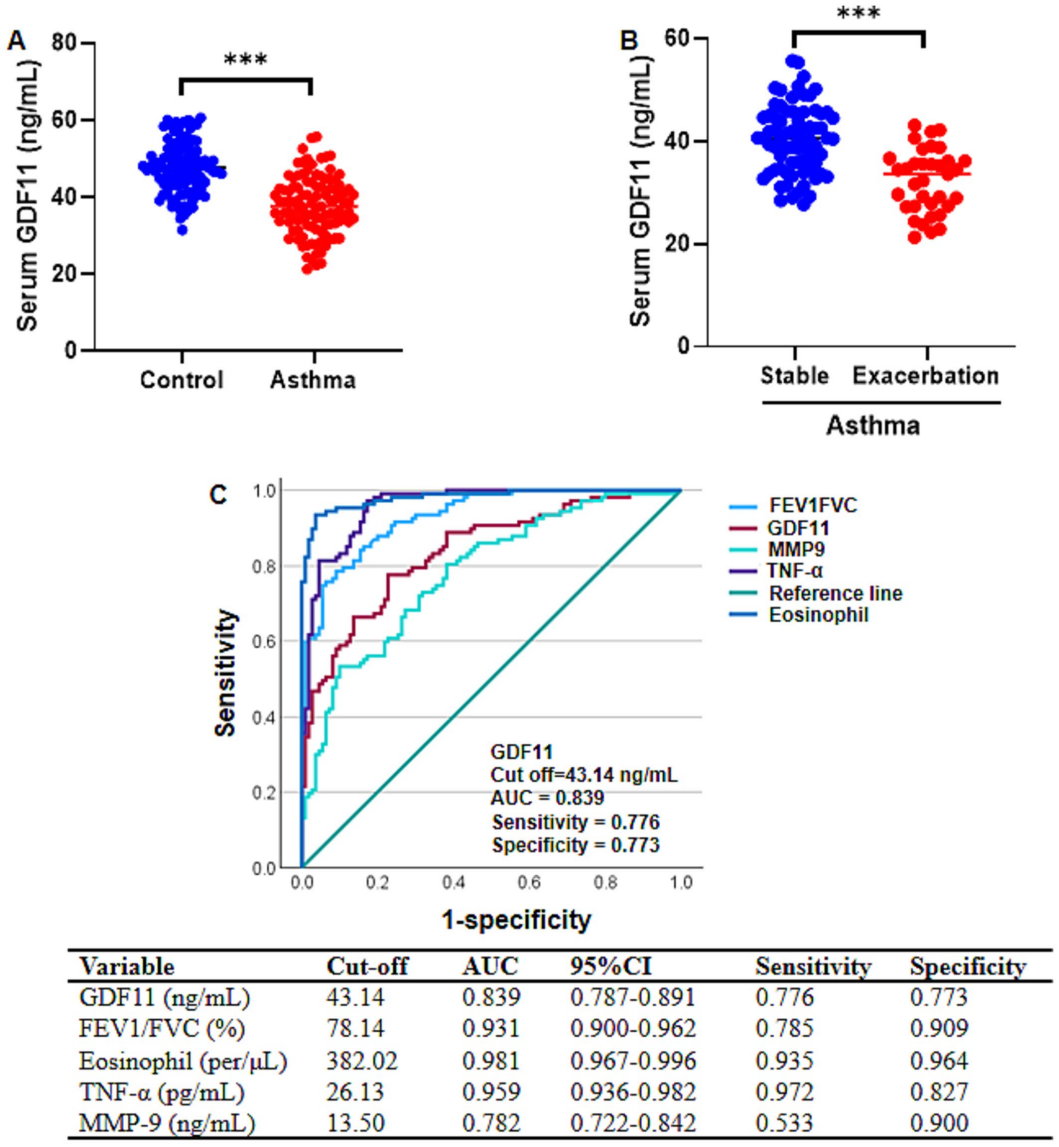
Serum GDF11 levels and diagnostic performance in asthma. **(A)** Comparison of serum GDF11 concentrations between healthy controls and patients with asthma, showing significantly reduced GDF11 levels in the asthma group. **(B)** Serum GDF11 levels in patients with stable asthma and those with asthma exacerbation, demonstrating a marked decrease during exacerbation. **(C)** Receiver operating characteristic (ROC) curve analysis comparing the diagnostic performance of GDF11 with FEV1/FVC, eosinophil count, TNF-*α*, and MMP-9 for identifying asthma exacerbation. The optimal cut-off value for GDF11 was 43.14 ng/mL, with an area under the curve (AUC) of 0.839, sensitivity of 77.6%, and specificity of 77.3%. Data are presented as individual values. ***p < 0.001.

Serum GDF11 concentrations were significantly lower in patients with asthma than in healthy controls (Figure 2A, *p* < 0.001). Among patients with asthma, those experiencing exacerbations exhibited markedly reduced serum GDF11 levels compared to those in a stable state (Figure 2B, *p* < 0.001). ROC curve analysis demonstrated that GDF11 had good discriminatory ability for identifying asthma exacerbation (AUC = 0.839, 95% CI: 0.787–0.891), with an optimal cut-off value of 43.14 ng/mL, yielding a sensitivity of 77.6% and specificity of 77.3% (Figure 2C). In comparison, FEV1/FVC, eosinophil count, and TNF-*α* showed excellent diagnostic performance (AUCs = 0.931, 0.981, and 0.959, respectively), whereas MMP-9 showed moderate accuracy (AUC = 0.782). Overall, these findings indicate that reduced serum GDF11 levels are associated with asthma severity and may serve as a useful biomarker for distinguishing asthma exacerbations, with a diagnostic performance comparable to that of established inflammatory and functional indicators.

### Association between serum GDF11 levels and pulmonary function in pediatric asthma

**Figure 3:**
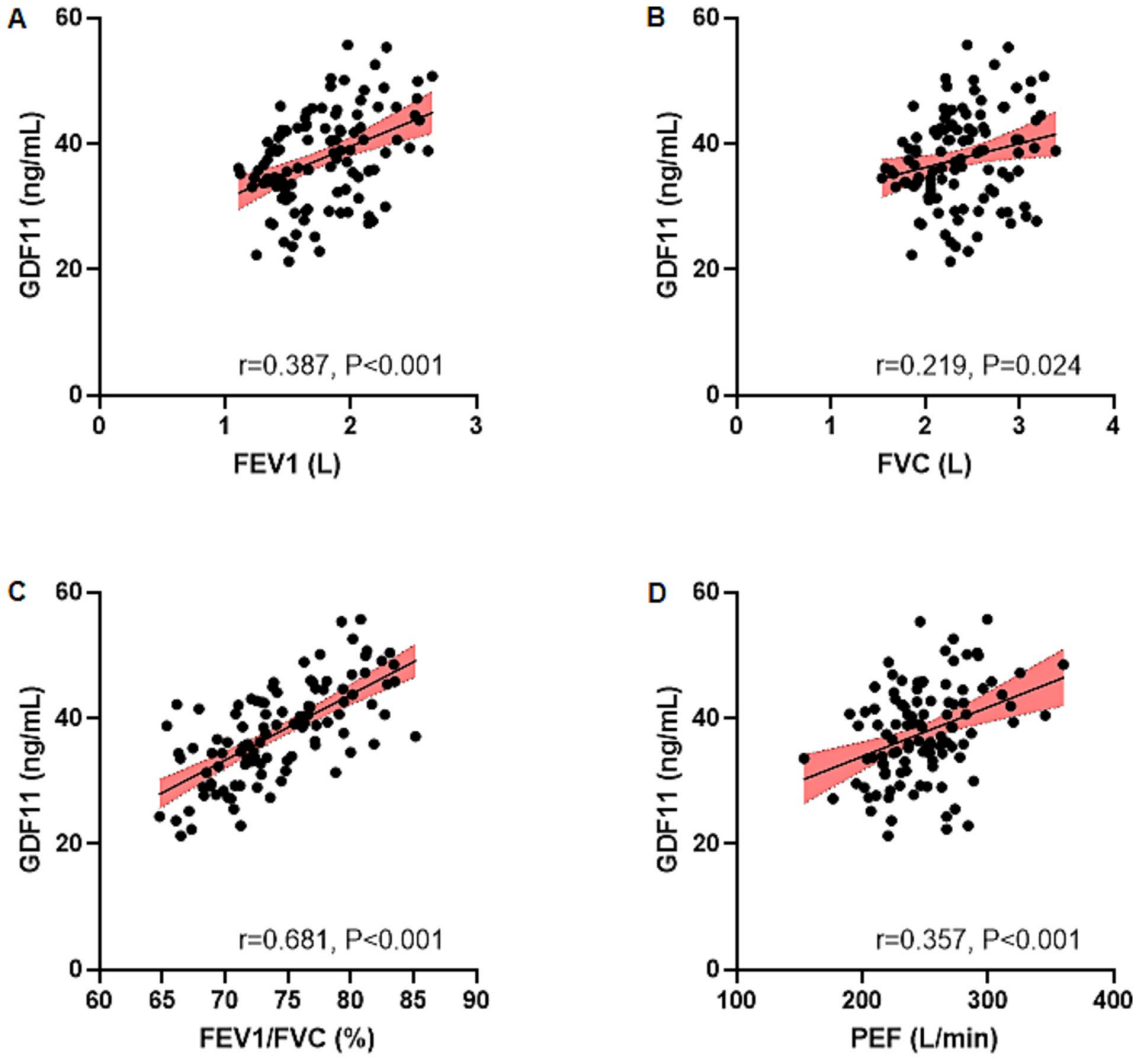
Correlation between serum GDF11 levels and pulmonary function parameters in pediatric patients with asthma. Serum GDF11 is positively correlated with **(A)** forced expiratory volume in one second (FEV1), **(B)** forced vital capacity (FVC), **(C)** FEV1/FVC, and **(D)** peak expiratory flow rate (PEF). Spearman’s rank correlation analysis was performed.

Correlation analysis revealed significant positive associations between serum GDF11 levels and lung function parameters in pediatric asthma patients (Figure 3). Serum GDF11 levels were moderately positively correlated with FEV1 (*r* = 0.387, *p* < 0.001) and were weakly but significantly correlated with FVC (*r* = 0.219, *p* = 0.024). A strong positive correlation was observed between GDF11 levels and FEV1/FVC ratio (*r* = 0.681, *p* < 0.001). In addition, serum GDF11 levels were positively correlated with PEF (*r* = 0.357, *p* < 0.001). These findings indicate that higher circulating GDF11 levels are associated with better pulmonary function in pediatric asthma, suggesting a potential protective or disease-modifying role for GDF11 in airway obstruction.

### Association between serum GDF11 levels and inflammatory parameters in pediatric asthma

**Figure 4:**
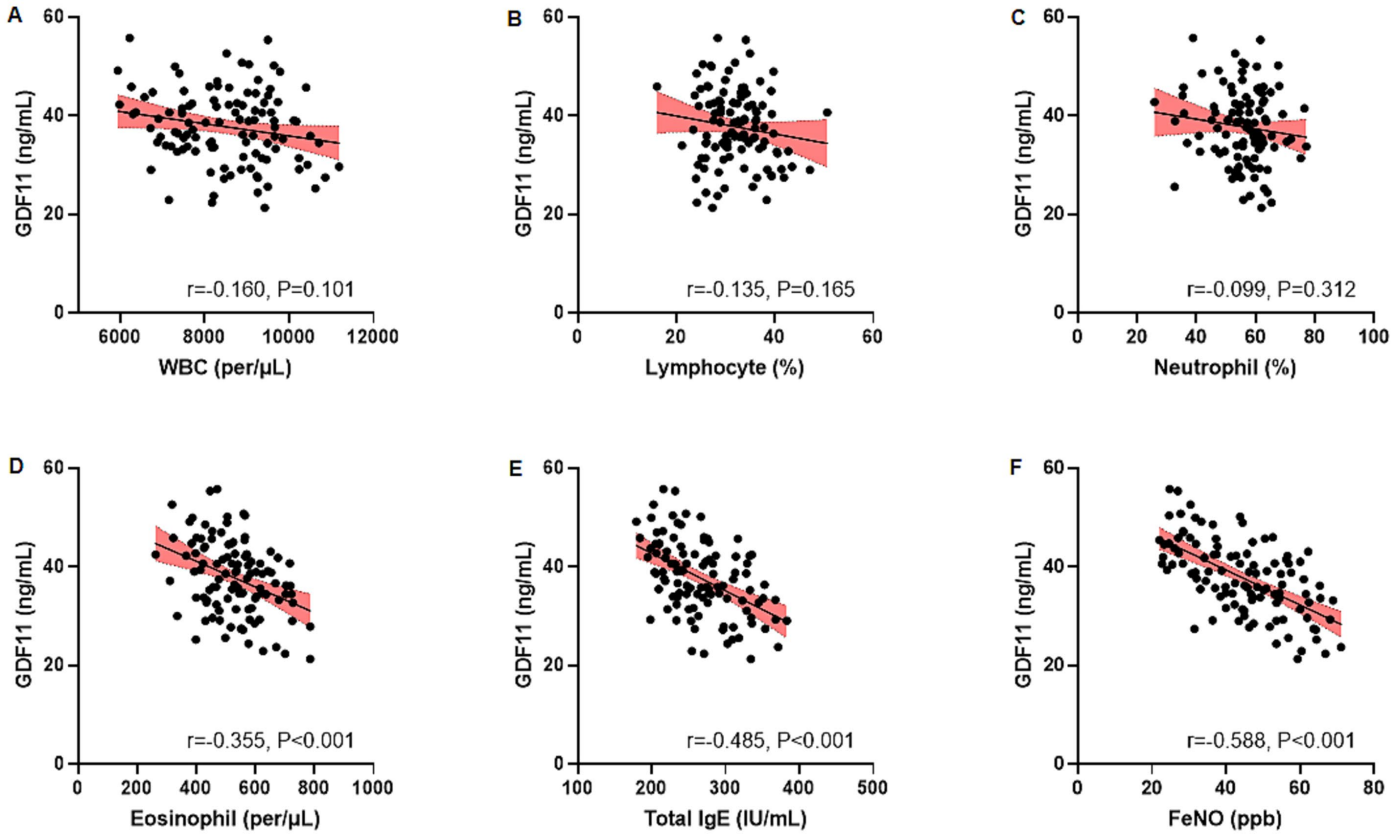
Correlation between serum GDF11 levels and blood routine and immune indicators in asthma pediatric patients. Serum GDF11 is negatively correlated with **(A)** WBC, **(B)** lymphocyte count, **(C)** neutrophil count, **(D)** eosinophil count, **(E)** Total IgE and **(F)** FeNO.

Correlation analyses revealed no significant associations between serum GDF11 levels and total white blood cell count, lymphocyte percentage, or neutrophil percentage (Figures 4A–C, all *p* > 0.05). In contrast, serum GDF11 levels were significantly and inversely correlated with key markers of type 2 airway inflammation (Figures 4D–F). Specifically, GDF11 levels were negatively correlated with blood eosinophil counts (*r* = −0.355, *p* < 0.001), total IgE levels (*r* = −0.485, *p* < 0.001), and FeNO levels (*r* = −0.588, *p* < 0.001). These findings indicate that lower circulating GDF11 levels are associated with enhanced eosinophilic inflammation and allergic airway responses in children with asthma.

### Correlation between serum GDF11 and inflammatory cytokines

**Figure 5:**
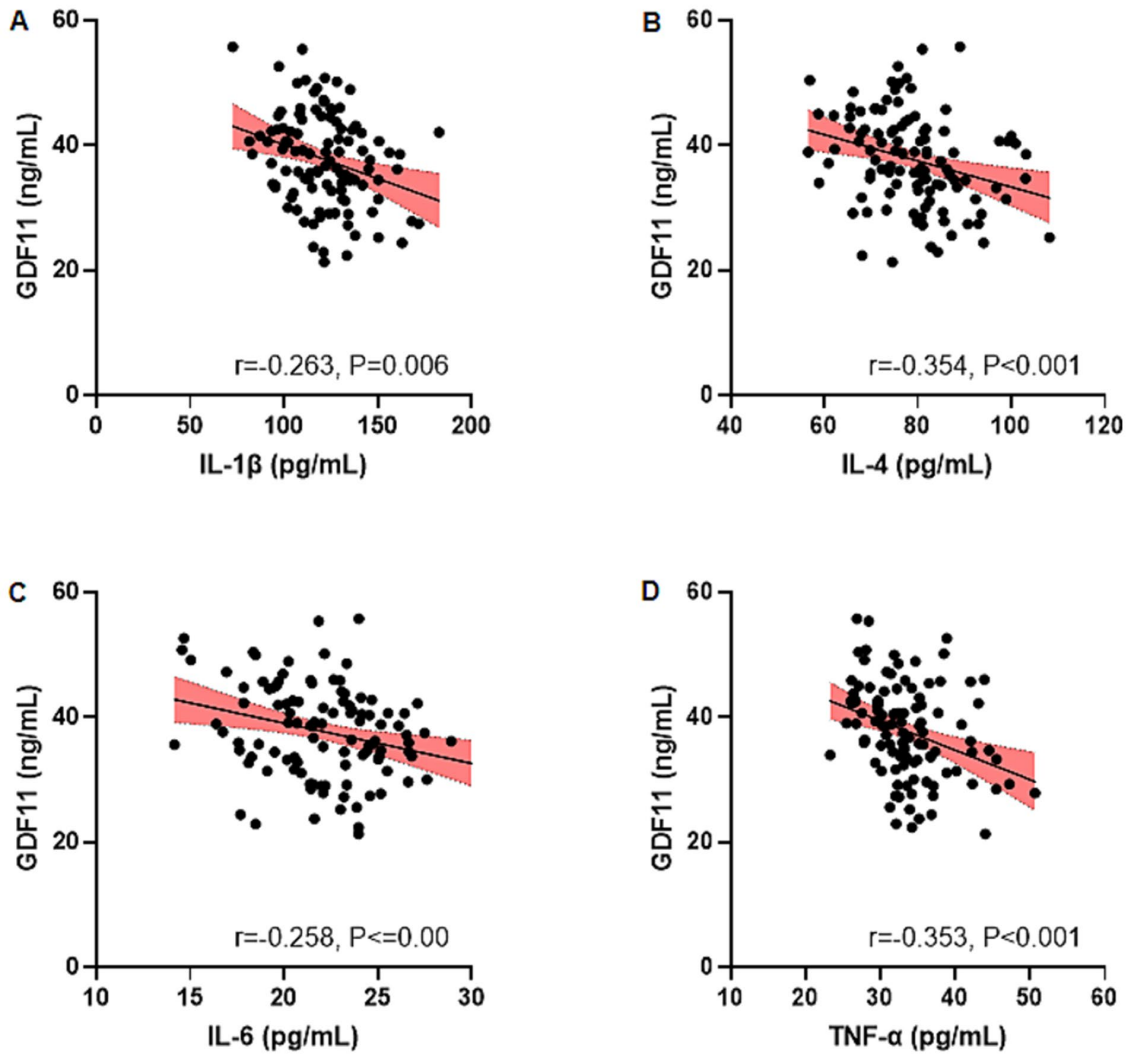
Correlation between serum GDF11 levels and inflammatory cytokines in asthma pediatric patients. Serum GDF11 is negatively correlated with **(A)** IL-1β, **(B)** IL-4, **(C)** IL-6, and **(D)** TNF-α.

Serum GDF11 levels were inversely correlated with the levels of multiple inflammatory cytokines in pediatric patients with asthma. As illustrated in Figure 5, GDF11 concentrations were significantly negatively associated with IL-1β (*r* = −0.263, *p* = 0.006), IL-4 (*r* = −0.354, *p* < 0.001), IL-6 (*r* = −0.258, *p* < 0.001), and TNF-*α* (*r* = −0.353, *p* < 0.001). These results indicate that elevated inflammatory cytokine levels are consistently associated with reduced circulating GDF11 levels, supporting the potential involvement of GDF11 in the inflammatory regulation of pediatric asthma.

### Association between serum GDF11 and airway remodeling markers

**Figure 6:**
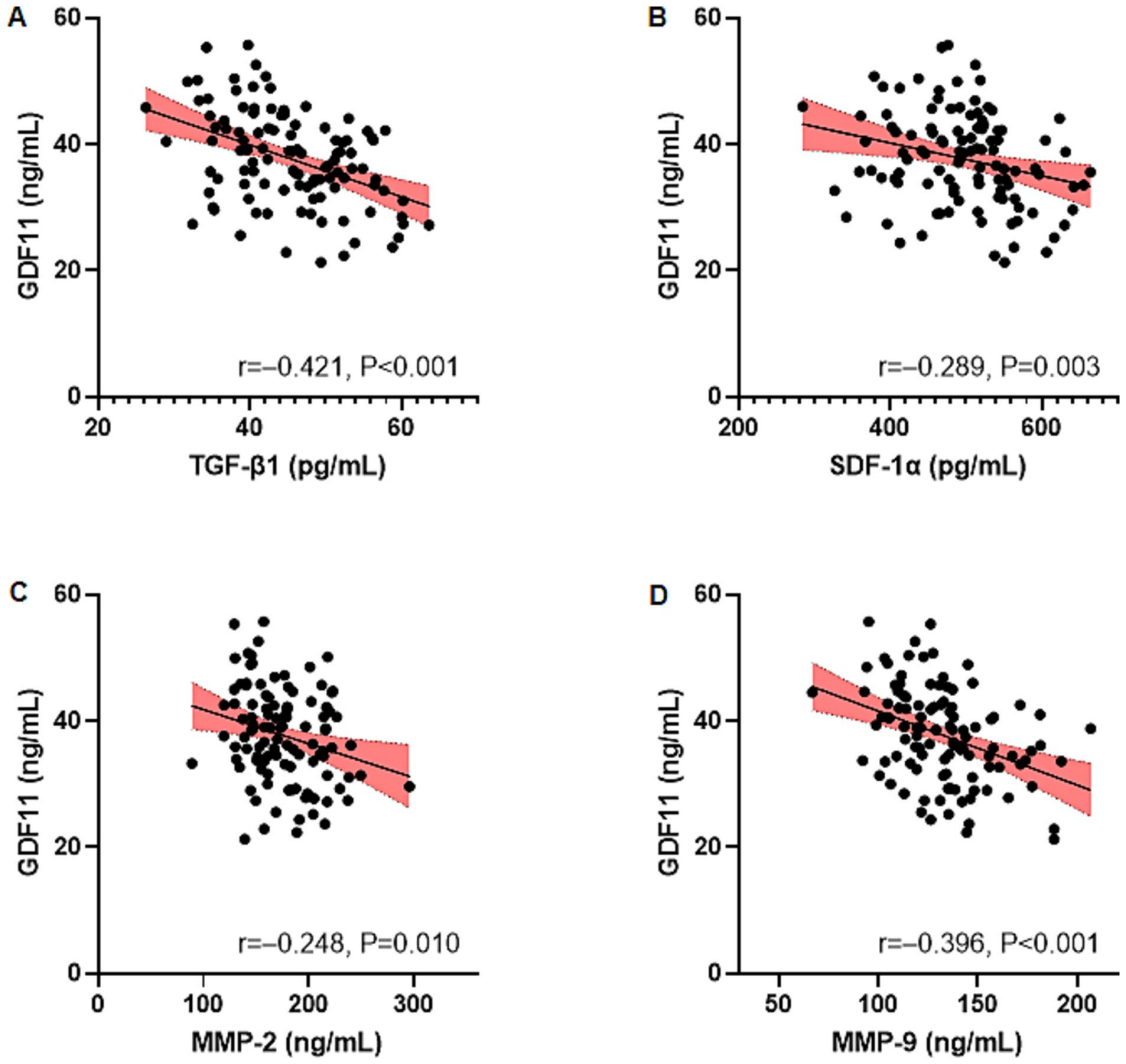
Correlation between serum GDF11 levels and serum airway remodeling markers in asthma pediatric patients. Serum GDF11 is negatively correlated with **(A)** TGF-β1, **(B)** SDF-1α, **(C)** MMP-2, and **(D)** MMP-9.

Serum GDF11 levels were significantly and inversely correlated with multiple airway remodeling-related biomarkers in pediatric patients with asthma. As shown in Figure 6, GDF11 concentrations were negatively associated with TGF-β1 (*r* = −0.421, *p* < 0.001), SDF-1α (*r* = −0.289, *p* = 0.003), MMP-2 (*r* = −0.248, *p* = 0.010), and MMP-9 (*r* = −0.396, *p* < 0.001) levels. These results indicate that lower circulating GDF11 levels are associated with enhanced profibrotic signaling and extracellular matrix remodeling, suggesting that circulating GDF11 levels are associated with markers of airway structural changes in children with asthma.

## Discussion

In this study, we demonstrated that circulating GDF11 levels are significantly reduced in pediatric patients with asthma, particularly during acute exacerbations, and are closely associated with airway inflammation, lung function impairment, and airway remodeling markers. These findings suggest that lower GDF11 levels are associated with asthma severity and exacerbation status and may serve as potential biomarkers of disease activity.

Asthma is a heterogeneous inflammatory airway disease characterized by variable airflow limitations, type 2-dominant inflammation, and progressive airway remodeling, particularly in children with poorly controlled disease or recurrent exacerbations. Consistent with previous reports, our asthma cohort exhibited significantly impaired pulmonary function, elevated eosinophil counts, increased IgE levels, and higher FeNO levels, reflecting enhanced eosinophilic and allergic inflammation (1, 6, 22). In parallel, we observed increased levels of proinflammatory and profibrotic mediators, including IL-1*β*, IL-4, IL-6, TNF-*α*, and TGF-β1, as well as remodeling-associated factors such as SDF-1α, MMP-2, and MMP-9, supporting the presence of active inflammatory and structural airway changes (10, 23, 24).

A novel and important finding of this study is the marked reduction in serum GDF11 levels in patients with asthma compared to healthy controls, with the lowest levels observed during acute exacerbations. GDF11, a member of the TGF-β superfamily, has been implicated in diverse biological processes, including embryonic development, tissue homeostasis, inflammation, and fibrosis (25, 26). Although early studies highlighted the potential rejuvenating effects of GDF11, recent evidence suggests that its biological actions are context-dependent and tissue-specific (11, 26). Our findings extend this concept to pediatric asthma, suggesting that reduced GDF11 levels are associated with increased airway inflammation and greater disease severity.

The inverse associations observed between GDF11 levels and eosinophil counts, IgE, and FeNO suggest a close relationship between GDF11 deficiencies and type 2 inflammatory responses. IL-4, a key cytokine driving Th2 polarization, IgE class switching, and eosinophilic inflammation, showed a strong negative correlation with GDF11. Similar inverse relationships were noted for IL-1β, IL-6, and TNF-*α*, which are known to contribute to airway hyper responsiveness, epithelial injury, and exacerbation severity (27–29). These findings indicate that GDF11 levels are inversely associated with inflammatory markers, suggesting a potential relationship between GDF11 and airway inflammatory status, although the directionality and underlying mechanisms of this relationship remain unknown.

Airway remodeling is a hallmark of chronic asthma that contributes to irreversible airflow limitations. TGF-*β*1 is a central mediator of subepithelial fibrosis, smooth muscle proliferation, and extracellular matrix deposition (10). In this study, GDF11 levels were inversely correlated with those of TGF-β1, SDF-1α, MMP-2, and MMP-9, all of which are critically involved in fibroblast activation, inflammatory cell recruitment, and matrix remodeling (30–32). These findings suggest that reduced circulating GDF11 levels are associated with increased profibrotic signaling and airway remodeling. Given that GDF11 and TGF-β share overlapping signaling pathways, this observation raises the possibility of a shared regulatory axis; however, the mechanism of involvement cannot be inferred from the present study (33, 34).

Importantly, multivariate logistic regression analysis identified lower serum GDF11 levels as being independently associated with an increased risk of asthma exacerbation, even after adjusting for lung function and inflammatory marker levels. This highlights the potential clinical relevance of GDF11 beyond traditional predictors, such as eosinophil counts and the FEV1/FVC ratio (35, 36). Furthermore, ROC curve analysis demonstrated that GDF11 has good diagnostic performance for distinguishing asthma exacerbations, with an accuracy comparable to that of established inflammatory and functional indices in the validation cohort. Although GDF11 alone may not outperform traditional biomarkers, its combination with lung function and inflammatory markers may improve the risk stratification and monitoring of pediatric asthma.

## Limitations

This study had several limitations. First, the cross-sectional design precluded causal inference regarding the role of GDF11 in asthma pathogenesis or progression. Although short-term follow-up (3–6 months) was available for some participants, these data were not collected in a standardized or complete manner, precluding reliable longitudinal or predictive analyses, including the assessment of whether baseline GDF11 levels predict future exacerbations or lung function decline. Second, serum GDF11 levels may not fully reflect local airway expression or activity, and mechanistic studies clarifying the role in inflammation and airway remodeling have not yet been performed. Third, although medication use can influence inflammatory biomarkers, this potential confounding effect was minimized by excluding patients receiving maintenance controller therapy or systemic glucocorticoids within the preceding 3 months. Nevertheless, residual confounding cannot be entirely ruled out, and future longitudinal studies should assess the effects of ongoing asthma treatment on circulating GDF11 levels. Fourth, although BMI did not differ significantly between the asthma and control groups, this similarity was not due to intentional BMI matching and may reflect the selection bias inherent in the study population. Given that childhood obesity is associated with specific asthma phenotypes, including more severe symptoms and altered inflammatory profiles, the generalizability of our findings to populations with a higher prevalence of obesity-related asthma may be limited. Therefore, future studies should include more diverse cohorts with a broader BMI distribution to further evaluate the potential interactions between adiposity, GDF11 levels, and asthma pathophysiology. Fifth, despite adjusting for major clinical and laboratory variables, several important potential confounders (e.g., atopic status, family history, prior exacerbations, and comorbid allergic conditions) were not systematically collected, leaving residual confounding. Finally, diagnostic heterogeneity related to age-dependent differences in asthma assessment may persist, particularly in younger children (≤5 years). Well-designed prospective longitudinal studies with standardized outcomes and mechanistic validation are required to further define the role of GDF11 in pediatric asthma.

## Conclusion

**Figure 7:**
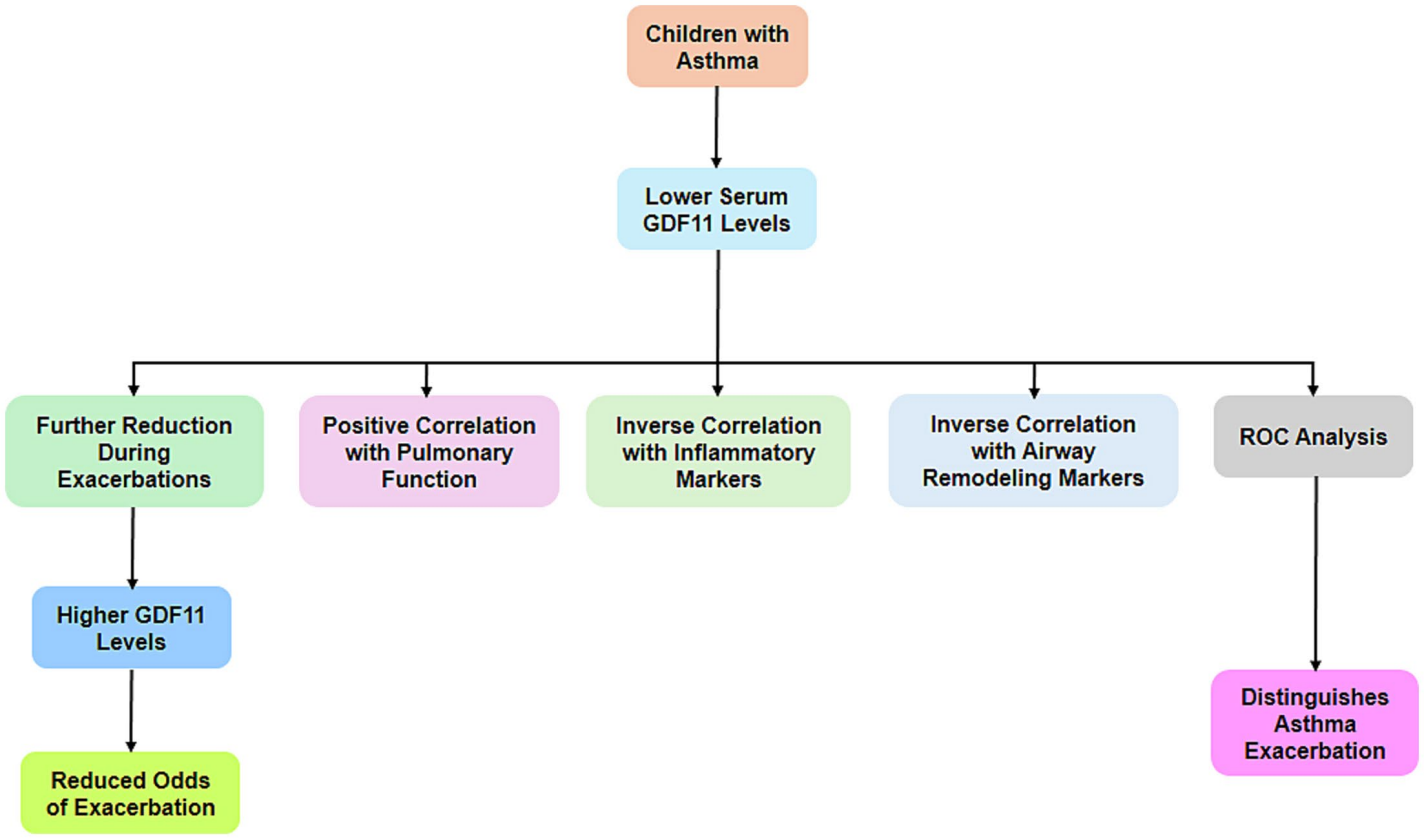
Schematic presentation of the clinical associations and predictive value of serum GDF11 in childhood asthma. Children with asthma exhibit reduced serum GDF11 levels, with a further decline observed during acute exacerbations. Higher serum GDF11 levels are associated with improved pulmonary function and reduced odds of asthma exacerbation. Serum GDF11 levels show inverse correlations with inflammatory markers and airway remodeling markers. Receiver operating characteristic (ROC) analysis demonstrates that serum GDF11 has potential discriminatory ability for identifying asthma exacerbations.

In conclusion, reduced serum GDF11 levels are associated with asthma exacerbations, impaired lung function, enhanced type 2 inflammation, and airway remodeling in children with asthma (Figure 7). These findings support the use of GDF11 as a potential biomarker associated with asthma severity and exacerbation risk. However, further longitudinal and mechanistic studies are required to determine whether GDF11 plays a causal or functional role in the pathophysiology of asthma.

## Data Availability

The datasets presented in this study can be found in online repositories. The names of the repository/repositories and accession number(s) can be found in the article/supplementary material.

## References

[1] Holgate ST. Pathogenesis of asthma. Clin Exp Allergy. (2008) 38:872–97. doi: 10.1111/j.1365-2222.2008.02971.x, 18498538

[2] Bergeron C Tulic MK Hamid Q. Airway remodelling in asthma: from benchside to clinical practice. Can Respir J. (2010) 17:e85–93. doi: 10.1155/2010/318029, 20808979 PMC2933777

[3] Kudo M Ishigatsubo Y Aoki I. Pathology of asthma. Front Microbiol. (2013) 4:263. doi: 10.3389/fmicb.2013.00263, 24032029 PMC3768124

[4] Saglani S Lloyd CM. Novel concepts in airway inflammation and remodelling in asthma. Eur Respir J. (2015) 46:1796–804. doi: 10.1183/13993003.01196-2014, 26541520

[5] Fahy JV. Type 2 inflammation in asthma-present in most, absent in many. Nat Rev Immunol. (2015) 15:57–65. doi: 10.1038/nri3786, 25534623 PMC4390063

[6] Halwani R Al-Muhsen S Al-Jahdali H Hamid Q. Role of transforming growth factor-β in airway remodeling in asthma. Am J Respir Cell Mol Biol. (2011) 44:127–33. doi: 10.1165/rcmb.2010-0027TR, 20525803

[7] Xu F Liu C Zhou D Zhang L. TGF-β/SMAD pathway and its regulation in hepatic fibrosis. J Histochem Cytochem. (2016) 64:157–67. doi: 10.1369/0022155415627681, 26747705 PMC4810800

[8] Panek M Pietras T Szemraj J Fabijan A Kuna P. Identification and association of TGFβ-1 expression in patients with asthma in a polish population - Lodz metropolitan area study. Int J Biochem Mol Biol. (2013) 4:67–74. 23638322 PMC3627069

[9] Hough KP Curtiss ML Blain TJ Liu RM Trevor J Deshane JS . Airway remodeling in asthma. Front Med. (2020) 7:191. doi: 10.3389/fmed.2020.00191, 32509793 PMC7253669

[10] Egerman MA Cadena SM Gilbert JA Meyer A Nelson HN Swalley SE . GDF11 increases with age and inhibits skeletal muscle regeneration. Cell Metab. (2015) 22:164–74. doi: 10.1016/j.cmet.2015.05.010, 26001423 PMC4497834

[11] Loffredo FS Steinhauser ML Jay SM Gannon J Pancoast JR Yalamanchi P . Growth differentiation factor 11 is a circulating factor that reverses age-related cardiac hypertrophy. Cell. (2013) 153:828–39. doi: 10.1016/j.cell.2013.04.015, 23663781 PMC3677132

[12] Walker RG Poggioli T Katsimpardi L Buchanan SM Oh J Wattrus S . Biochemistry and biology of GDF11 and Myostatin: similarities, differences, and questions for future investigation. Circ Res. (2016) 118:1125–42. doi: 10.1161/CIRCRESAHA.116.308391, 27034275 PMC4818972

[13] Vanhoutte D Schips TG Minerath RA Huo J Kavuri NSS Prasad V . Thbs1 regulates skeletal muscle mass in a TGFβ-Smad2/3-ATF4-dependent manner. Cell Rep. (2024) 43:114149. doi: 10.1016/j.celrep.2024.114149, 38678560 PMC11217783

[14] Andersson O Reissmann E Jörnvall H Ibáñez CF. Synergistic interaction between Gdf1 and nodal during anterior axis development. Genes Dev. (2006) 293:370–81. doi: 10.1016/j.ydbio.2006.02.002, 16564040

[15] Sinha M Jang YC Oh J Khong D Wu EY Manohar R . Restoring systemic GDF11 levels reverses age-related dysfunction in mouse skeletal muscle. Science. (2014) 344:649–52. doi: 10.1126/science.1251152, 24797481 PMC4104429

[16] Meng XM Nikolic-Paterson DJ Lan HY. TGF-β: the master regulator of fibrosis. Nat Rev Nephrol. (2016) 12:325–38. doi: 10.1038/nrneph.2016.48, 27108839

[17] Machelak W Szczepaniak A Jacenik D Zielińska M. The role of GDF11 during inflammation - an overview. Life Sci. (2023) 322:121650. doi: 10.1016/j.lfs.2023.121650, 37011872

[18] Li Q Li H Zhu L Zhang L Zheng X Hao Z. Growth differentiation factor 11 evokes lung injury, inflammation, and fibrosis in mice through the Activin a receptor type II-like kinase, 53kDa-Smad2/3 signaling pathway. Am J Pathol. (2024) 194:2036–58. doi: 10.1016/j.ajpath.2024.07.016, 39147236

[19] Hong J Bao Y Chen A Li C Xiang L Liu C . Chinese guidelines for childhood asthma 2016: major updates, recommendations and key regional data. J Asthma. (2016) 55:1138–46. doi: 10.1080/02770903.2017.1396474, 29227721

[20] Menzies D Nair A Lipworth BJ. Portable exhaled nitric oxide measurement: comparison with the "gold standard" technique. Chest. (2007) 131:410–4. doi: 10.1378/chest.06-1335, 17296641

[21] Global Initiative for Asthma (GINA) (2024). Global Strategy for Asthma Management and Prevention. Fontana, Wisconsin, USA: Global Initiative for Asthma (GINA).

[22] Walsh GM. Novel cytokine-directed therapies for asthma. Discov Med. (2011) 11:283–91. 21524382

[23] Lambrecht BN Ahmed E Hammad H. The immunology of asthma. Nat Immunol. (2025) 26:1233–45. doi: 10.1038/s41590-025-02212-9, 40730897

[24] McPherron AC. Metabolic functions of myostatin and gdf11. Immunol Endocr Metab Agents Med Chem. (2010) 10:217–31. doi: 10.2174/187152210793663810, 21197386 PMC3011861

[25] Ma Y Liu Y Han F Qiu H Shi J Huang N . Growth differentiation factor 11: a "rejuvenation factor" involved in regulation of age-related diseases? Aging. (2021) 13:12258–72. doi: 10.18632/aging.202881, 33886503 PMC8109099

[26] Lemanske RF Jr Busse WW. Asthma: clinical expression and molecular mechanisms. J Allergy Clin Immunol. (2010) 125:S95–S102. doi: 10.1016/j.jaci.2009.10.047, 20176271 PMC2853245

[27] Lambrecht BN Hammad H Fahy JV. The cytokines of asthma. Immunity. (2019) 50:975–91. doi: 10.1016/j.immuni.2019.03.018, 30995510

[28] Chung KF. Inflammatory biomarkers in severe asthma. Curr Opin Pulm Med. (2012) 18:35–41. doi: 10.1097/MCP.0b013e32834d09a5, 22045348

[29] Hirota N Martin JG. Mechanisms of airway remodeling. Chest. (2013) 144:1026–32. doi: 10.1378/chest.12-3073, 24008953

[30] Mo B Li J Wei J Wang C Zeng J Wang J . The role of SDF-1/CXCR4 on airway inflammation and airway remodeling in a rat asthma model. Zhonghua Jie He He Hu Xi Za Zhi. (2015) 38:39–44. 25791655

[31] Bajbouj K Ramakrishnan RK Hamid Q. Role of matrix metalloproteinases in angiogenesis and its implications in asthma. J Immunol Res. (2021) 2021:1–12. doi: 10.1155/2021/6645072, 33628848 PMC7896871

[32] Frohlich J Vinciguerra M. Candidate rejuvenating factor GDF11 and tissue fibrosis: friend or foe? Geroscience. (2020) 42:1475–98. doi: 10.1007/s11357-020-00279-w, 33025411 PMC7732895

[33] Massagué J. TGFβ signalling in context. Nat Rev Mol Cell Biol. (2012) 13:616–30. doi: 10.1038/nrm3434, 22992590 PMC4027049

[34] Price DB Rigazio A Campbell JD Bleecker ER Corrigan CJ Thomas M . Blood eosinophil count and prospective annual asthma disease burden: a UK cohort study. Lancet Respir Med. (2015) 3:849–58. doi: 10.1016/S2213-2600(15)00367-7, 26493938

[35] Killane I Sulaiman I MacHale E Breathnach A Taylor TE Holmes MS . Predicting asthma exacerbations employing remotely monitored adherence. Healthc Technol Lett. (2016) 3:51–5. doi: 10.1049/htl.2015.0058, 27222733 PMC4814807

[36] Hammad H Lambrecht BN. The basic immunology of asthma. Cell. (2021) 184:1469–85. doi: 10.1016/j.cell.2021.02.016, 33711259

